# Supplementary material for: LOCUS (LOng Covid–Understanding Symptoms, events and use of services in Portugal): A three-component study protocol
Source: PLoS One. 2023 Apr 26;18(4):e0285051. doi: 10.1371/journal.pone.0285051 (PMC10132590; doi:10.1371/journal.pone.0285051)
Supplement: S1 Table — (DOCX) [file pone.0285051.s001.docx]

**Supporting information – S1 Table**

**S1A Table.** Data dictionary (“Cardiovascular and respiratory events following COVID-19” component).

| **Variable_name** | **Label** | **Type** | **Values** |
| --- | --- | --- | --- |
| **A1. Participant characterization** | | | |
| ID | Participant ID | Numeric |  |
| begin_quest | Questionnaire start time | Date |  |
| end_quest | Questionnaire end time | Date |  |
| time_quest | Duration of questionnaire | Numeric |  |
| Hospital_ID | Hospital ID | Factor |  |
| death | Did the participant die after hospital discharge? | Factor | 0 - No 1 - Yes |
| days_death SHOW ONLY IF [death=1] | Number of days between positive test and the participants death | Numeric |  |
| COVID_death SHOW ONLY IF [death=1] | Cause of death | Factor | 1 - COVID-19 2 - Non-COVID-19 8 - Unknown NA - Not applicable |
| cause_death SHOW ONLY IF [death=1] | Cause of death | Text |  |
| end_time SHOW ONLY IF [death!=1] | Number of days between positive test and loss to follow-up | Numeric |  |
| sex | Participant sex | Factor | 1 - Men 2 - Women |
| age | Participant age at the positive test | Numeric |  |
| smoking | Smoking status | Factor | 1 - Smoker 2 - Former 3 - Never 8 - Unknown |
| years_stop SHOW ONLY IF [smoking=2] | How many years ago has the participant stopped smoking? | Numeric | 99999 - Unknown |
| pack_year SHOW ONLY IF [smoking=1] | Number of packs per year before the positive test | Numeric | 99999 - Unknown |
| alcohol | Participant drinks more than 12g of ethanol before the positive test | Factor | 0 - No 1 - Yes 8 - Unknown |
| exercise | Participant exercises 30 minutes or more daily and 3 or more time weekly before the positive test | Factor | 0 - No 1 - Yes 8 - Unknown |
| **A2. Clinical status before COVID-19** | | | |
| base_hypertension | Previous hypertension | Factor | 0 - No 1 - Yes |
| base_stroke | Previous hemorrhagic/ischemic stroke | Factor | 0 - No 1 - Yes |
| base_ischemic | Previous ischemic heart disease | Factor | 0 - No 1 - Yes |
| base_heart_failure | Previous heart failure | Factor | 0 - No 1 - Yes |
| base_myocarditis | Previous myocarditis | Factor | 0 - No 1 - Yes |
| base_atrial_fib | Previous atrial fibrillation | Factor | 0 - No 1 - Yes |
| base_other_arrhythmias | Previous other arrhythmias | Factor | 0 - No 1 - Yes |
| base_myocard_infarc | Previous acute myocardial infarction | Factor | 0 - No 1 - Yes |
| base_intra_thrombus | Previous intracardiac thrombus | Factor | 0 - No 1 - Yes |
| base_COPD | Previous chronic obstructive pulmonary disease | Factor | 0 - No 1 - Yes |
| base_asthma | Previous asthma | Factor | 0 - No 1 - Yes |
| base_resp_failure | Previous chronic respiratory failure | Factor | 0 - No 1 - Yes |
| base_pulm_thrombo | History of pulmonary thromboembolism | Factor | 0 - No 1 - Yes |
| base_pulm_fib | Previous pulmonary fibrosis/Interstitial lung disease | Factor | 0 - No 1 - Yes |
| base_bronchiectasis | Previous bronchiectasis | Factor | 0 - No 1 - Yes |
| base_obesity | Previous obesity (BMI≥30) | Factor | 0 - No 1 - Yes |
| base_diabetes | Previous type 1 or 2 diabetes mellitus | Factor | 0 - No 1 - Yes |
| base_dyslipidemia | Previous dyslipidemia | Factor | 0 - No 1 - Yes |
| base_autoimmune | Previous autoimmune disease under immunosuppressant/immunomodulator | Factor | 0 - No 1 - Yes |
| base_psychiatric | Previous psychiatric disorder | Factor | 0 - No 1 - Yes |
| base_hypothyroidism | Previous hypothyroidism | Factor | 0 - No 1 - Yes |
| base_hyperthyroidism | Previous hyperthyroidism | Factor | 0 - No 1 - Yes |
| base_HIV | Previous type 1 or 2 HIV | Factor | 0 - No 1 - Yes |
| base_kidney | Previous chronic kidney disease | Factor | 0 - No 1 - Yes |
| base_thrombosis | History of deep vein thrombosis | Factor | 0 - No 1 - Yes |
| base_cirrhosis | Previous chronic liver disease/liver cirrhosis | Factor | 0 - No 1 - Yes |
| base_hepB | Previous hepatitis B | Factor | 0 - No 1 - Yes |
| base_hepC | Previous hepatitis C | Factor | 0 - No 1 - Yes |
| base_hema_neo | Previous active hematologic malignancy or history of hematologic malignancy diagnosed for less than 5 years and/or under treatment (last dose less than 1 year) | Factor | 0 - No 1 - Yes |
| base_solid_neo | Previous active solid organ neoplasm or history of solid organ neoplasm diagnosed at least 5 years ago and/or under treatment (last dose less than 1 year ago), and/or metastasis | Factor | 0 - No 1 - Yes |
| base_immuno_treat | Previous use of immunosuppressants/immunomodulators for less than a year | Factor | 0 - No 1 - Yes |
| base_anticoagulant | Previous use of anticoagulant therapy for less than a year | Factor | 0 - No 1 - Yes |
| **A3. Characterization of the disease and hospitalization** | | | |
| hosp_days | Number of days between positive test and hospitalization | Numeric |  |
| discharge_days | Number of days between positive test and discharge | Numeric |  |
| ward | Ward hospitalization | Factor | 0 - No 1 - Yes |
| days_ward SHOW ONLY IF [ward=1] | Number of days in ward | Numeric |  |
| ICU | Intensive care unit hospitalization | Factor | 0 - No 1 - Yes |
| days_ICU SHOW ONLY IF [ICU=1] | Number of days in ICU | Numeric |  |
| nosocomial | Nosocomial infection during hospitalization | Factor | 0 - No 1 - Yes |
| days_nosocomial SHOW ONLY IF [nosocomial=1] | Number of days between positive test and nosocomial infection | Numeric |  |
| nosocomial_resp SHOW ONLY IF [nosocomial=1] | Respiratory nosocomial infection | Factor | 0 - No 1 - Yes |
| nosocomial_urinary SHOW ONLY IF [nosocomial=1] | Urinary nosocomial infection | Factor | 0 - No 1 - Yes |
| nosocomial_gastro SHOW ONLY IF [nosocomial=1] | Gastrointestinal nosocomial infection | Factor | 0 - No 1 - Yes |
| nosocomial_cardiac SHOW ONLY IF [nosocomial=1] | Cardiac nosocomial infection | Factor | 0 - No 1 - Yes |
| nosocomial_gyn SHOW ONLY IF [nosocomial=1] | Gynecological nosocomial infection | Factor | 0 - No 1 - Yes |
| nosocomial_bone SHOW ONLY IF [nosocomial=1] | Bone nosocomial infection | Factor | 0 - No 1 - Yes |
| nosocomial_hema SHOW ONLY IF [nosocomial=1] | Hematologic nosocomial infection | Factor | 0 - No 1 - Yes |
| nosocomial_CNS SHOW ONLY IF [nosocomial=1] | Central nervous system nosocomial infection | Factor | 0 - No 1 - Yes |
| nosocomial_oto SHOW ONLY IF [nosocomial=1] | Otolaryngology nosocomial infection | Factor | 0 - No 1 - Yes |
| nosocomial_unknown SHOW ONLY IF [nosocomial=1] | Unknown type of nosocomial infection | Factor | 0 - No 1 - Yes |
| bact_superinf | Bacterial superinfection (infection present on the date of hospital admission and/or in the first 48 hours) | Factor | 0 - No 1 - Yes |
| hydro | Hydroxychloroquine during hospitalization | Factor | 0 - No 1 - Yes |
| hydro_days SHOW ONLY IF [hydro=1] | Number of days of hydroxycloroquine treatment | Numeric | 99999 - Unknown |
| azithro | Azithromycin during hospitalization | Factor | 0 - No 1 - Yes |
| azithro_days SHOW ONLY IF [azithro=1] | Number of days of azithromycin treatment | Numeric | 99999 - Unknown |
| remdesivir | Remdesivir during hospitalization | Factor | 0 - No 1 - Yes |
| remdesivir_days SHOW ONLY IF [remdesivir=1] | Number of days of remdesivir treatment | Numeric | 99999 - Unknown |
| dexa | Dexamethasone during hospitalization | Factor | 0 - No 1 - Yes |
| dexa_days SHOW ONLY IF [dexa=1] | Number of days of dexamethasone treatment | Numeric | 99999 - Unknown |
| tocili | Tocilizumab during hospitalization | Factor | 0 - No 1 - Yes |
| tocili_days SHOW ONLY IF [tocili=1] | Number of days of tocilizumab treatment | Numeric | 99999 - Unknown |
| oxygen | Oxygen during hospitalization | Factor | 0 - No 1 - Yes |
| oxygen_days SHOW ONLY IF [oxygen=1] | Number of days of oxygen treatment | Numeric | 99999 - Unknown |
| PaO2_FiO2 SHOW ONLY IF [oxygen=1] | Worse PaO2/FiO2 ratio | Numeric | 99999 - Unknown |
| high_nasal SHOW ONLY IF [oxygen=1] | High flow nasal oxygen therapy during hospitalization | Factor | 0 - No 1 - Yes |
| high_nasal_days SHOW ONLY IF [high_nasal=1] | Number of days of high flow nasal oxygen therapy | Numeric | 99999 - Unknown |
| BIPAP SHOW ONLY IF [oxygen=1] | Non-invasive ventilation – BIPAP/CPAP during hospitalization | Factor | 0 - No 1 - Yes |
| BIPAP_days SHOW ONLY IF [BIPAP=1] | Number of days of non-invasive ventilation – BIPAP/CPAP | Numeric | 99999 - Unknown |
| mech_vent SHOW ONLY IF [oxygen=1] | Invasive mechanical ventilation during hospitalization | Factor | 0 - No 1 - Yes |
| mech_vent_days SHOW ONLY IF [mech_vent=1] | Number of days of invasive mechanical ventilation | Numeric | 99999 - Unknown |
| physical_rehab | Physical rehabilitation at the time of hospital discharge again (not present prior to hospitalization) | Factor | 0 - No 1 - Yes |
| long_oxygen | Long term oxygen therapy at the time of hospital discharge again (not present prior to hospitalization) | Factor | 0 - No 1 - Yes |
| **A4. Post-discharge clinical status** | | | |
| heart_failure | Heart failure after discharge, neither present during hospitalization nor described at the time of discharge | Factor | 0 - No 1 - Yes |
| heart_failure_days SHOW ONLY IF [heart_failure=1] | Number of days between positive test and heart failure | Numeric |  |
| myocarditis | Myocarditis after discharge, neither present during hospitalization nor described at the time of discharge | Factor | 0 - No 1 - Yes |
| myocarditis_days SHOW ONLY IF [myocarditis=1] | Number of days between positive test and myocarditis | Numeric |  |
| arrhythmia | Arrhythmia after discharge, neither present during hospitalization nor described at the time of discharge | Factor | 0 - No 1 - Yes |
| arrhythmia_days SHOW ONLY IF [arrhythmia=1] | Number of days between positive test and arrhythmia | Numeric |  |
| myocard_infarc | Acute myocardial infarction after discharge, neither present during hospitalization nor described at the time of discharge | Factor | 0 - No 1 - Yes |
| myocard_infarc_days SHOW ONLY IF [myocard_infarc=1] | Number of days between positive test and acute myocardial infarction | Numeric |  |
| pulm_thrombo | Pulmonary thromboembolism after discharge, neither present during hospitalization nor described at the time of discharge | Factor | 0 - No 1 - Yes |
| pulm_thrombo_days SHOW ONLY IF [pulm_thrombo=1] | Number of days between positive test and pulmonary thromboembolism | Numeric |  |
| intra_thrombus | Intracardiac thrombus after discharge, neither present during hospitalization nor described at the time of discharge | Factor | 0 - No 1 - Yes |
| intra_thrombus_days SHOW ONLY IF [intra_thrombus=1] | Number of days between positive test and intracardiac thrombus | Numeric |  |
| thrombosis | Deep vein thrombosis after discharge, neither present during hospitalization nor described at the time of discharge | Factor | 0 - No 1 - Yes |
| thrombosis_days SHOW ONLY IF [thrombosis=1] | Number of days between positive test and deep vein thrombosis | Numeric |  |
| pulm_fib | Pulmonary fibrosis after discharge, neither present during hospitalization nor described at the time of discharge | Factor | 0 - No 1 - Yes |
| pulm_fib_days SHOW ONLY IF [pulm_fib=1] | Number of days between positive test and pulmonary fibrosis | Numeric |  |
| resp_failure | Chronic or prolonged respiratory failure after discharge, neither present during hospitalization nor described at the time of discharge | Factor | 0 - No 1 - Yes |
| resp_failure_days SHOW ONLY IF [resp_failure=1] | Number of days between positive test and chronic or prolonged respiratory failure | Numeric |  |
| obs_rest_resp | Obstructive/restrictive lung disease after discharge, neither present during hospitalization nor described at the time of discharge | Factor | 0 - No 1 - Yes |
| obs_rest_resp_days SHOW ONLY IF [obs_rest_resp=1] | Number of days between positive test and obstructive/restrictive lung disease | Numeric |  |
| org_pneu | Organizing pneumonia after discharge, neither present during hospitalization nor described at the time of discharge | Factor | 0 - No 1 - Yes |
| org_pneu_days SHOW ONLY IF [org_pneu=1] | Number of days between positive test and organizing pneumonia | Numeric |  |
| ischemic_stroke | Ischemic stroke after discharge, neither present during hospitalization nor described at the time of discharge | Factor | 0 - No 1 - Yes |
| ischemic_stroke_days SHOW ONLY IF [ischemic_stroke=1] | Number of days between positive test and ischemic stroke | Numeric |  |
| hemorrhagic_stroke | Hemorrhagic heart attack after discharge, neither present during hospitalization nor described at the time of discharge | Factor | 0 - No 1 - Yes |
| hemorrhagic_stroke_days SHOW ONLY IF [hemorrhagic_stroke=1] | Number of days between positive test and hemorrhagic stroke | Numeric |  |
| other_event | Other relevant event after discharge, neither present during hospitalization nor described at the time of discharge | Factor | 0 - No 1 - Yes |
| other_event_descr SHOW ONLY IF [other_event=1] | Description of the other relevant event after discharge | Text |  |
| other_event_days SHOW ONLY IF [other_event=1] | Number of days between positive test and other relevant event | Numeric |  |
| **A5. Vaccination** | | | |
| flu_2019 | Received a flu vaccine in 2019 | Factor | 0 - No 1 - Yes |
| flu_2020 | Received a flu vaccine in 2020 | Factor | 0 - No 1 - Yes |
| flu_2021 | Received a flu vaccine in 2021 | Factor | 0 - No 1 - Yes |
| flu_2022 | Received a flu vaccine in 2022 | Factor | 0 - No 1 - Yes |
| flu_no | Did not receive a flu vaccination | Factor | 0 - No 1 - Yes |
| antipneumoc | Received the antipneumococcal vaccine in the last 5 years before the positive test | Factor | 1 - 13 valent 2 - 23 valent 3 - Both 4 - None |
| COVID_vac | Received any vaccine against COVID-19 | Factor | 0 - No 1 - Yes |
| time_first_vac SHOW ONLY IF [COVID_vac=1] | First vaccine against COVID-19 before the positive test | Factor | 0 - No 1 - Yes NA - Not applicable |
| first_after_days SHOW ONLY IF [time_first_vac=0] | Number of days between positive test and first dose or single dose | Numeric |  |
| first_before_days SHOW ONLY IF [time_first_vac=1] | Number of days between first dose or single dose and positive test | Numeric |  |
| time_second_vac SHOW ONLY IF [COVID_vac=1] | Second vaccine against COVID-19 before the positive test | Factor | 0 - No 1 - Yes 2 - Single dose 3 - Has not received yet or has not received  NA - Not applicable |
| second_after_days SHOW ONLY IF [time_second_vac=0] | Number of days between positive test and second dose | Numeric |  |
| second_before_days SHOW ONLY IF [time_second_vac=1] | Number of days between second dose and positive test | Numeric |  |
| time_reinf_vac SHOW ONLY IF [COVID_vac=1] | Reinforcement vaccine against COVID-19 before the positive test | Factor | 0 - No 1 - Yes 2 - Has not received yet or has not received  NA - Not applicable |
| reinf_after_days SHOW ONLY IF [time_reinf_vac=0] | Number of days between positive test and reinforcement dose | Numeric |  |
| reinf_before_days SHOW ONLY IF [time_reinf_vac=1] | Number of days between reinforcement dose and positive test | Numeric |  |
| observat | Observations | Text |  |

**S1B Table.** Data dictionary (“Physical and mental symptoms following COVID-19” component).

| **Variable_name** | **Label** | **Type** | **Values** |
| --- | --- | --- | --- |
| **B1. Participant characterization** | | | |
| ID | Participant ID | Numeric |  |
| begin_quest | Questionnaire start time | Date |  |
| end_quest | Questionnaire end time | Date |  |
| time_quest | Duration of questionnaire | Numeric |  |
| timepoint | Questionnaire period | Factor | 1 - 9 months 2 - 12 months |
| covid_test | Result of the RT-PCR COVID-19 test | Factor | 1 - Positive 2 - Negative |
| sex | Participant sex | Factor | 1 - Men 2 - Women 3 - Other |
| sex_other | Participant sex - open option for other | Text |  |
| age | Participant age at the positive test | Numeric |  |
| county | Municipality of residence | Factor |  |
| education | Participant's highest education level | Factor | 1 - Cannot read or write 2 - Does not have 1st cycle (4th year) 3 - 1st cycle (4th year) 4 - 2nd cycle (6th year)  5 - 3rd cycle (9th year)  6 - Secondary education (12th grade)  7 - Higher education |
| current_job | Participant's current occupation | Factor | 1 - Worker 2 - Unemployed 3 - Student 4 - Working student 5 - Retired 6 - Domestic worker/informal caregiver 7 - Incapacitated  8 - Another |
| change_hours SHOW ONLY IF [current_job=1 \| current_job=3 \| current_job=4] | Change in duration, in hours of work or school, compared to the situation they were previously in | Factor | 0 - No 1 - Yes |
| status_job SHOW ONLY IF [change_hours=1] | Output of the work or school change compared to the situation they were previously in | Factor | 1 - Increase 2 - Decrease 3 - Had to stop 88 - Unknown |
| reason_reduction SHOW ONLY IF [status_job=2 \| status_job=3] | Reason for the decrease or need to stop work or school | Factor | 1 - Poor health 2 - New carer status 3 - Restrictions due to pandemic COVID-19 4 - Other |
| other_reason SHOW ONLY IF [reason_reduction=4] | Other reason for the decrease or need to stop work or school | Text |  |
| smoking | Smoking status at the time of the RT-PCR COVID-19 test | Factor | 0 - No 1 - Yes |
| smoke_day SHOW ONLY IF [smoking=1] | Number of cigars smoked per day before the RT-PCR COVID-19 test | Numeric | 99999 - Unknown |
| smoke_age SHOW ONLY IF [smoking=1] | Age the participant started to smoke | Numeric | 99999 - Unknown |
| alcohol | Frequency of ingested alcoholic beverages | Factor | 1 - Never  2 - Once a month or less 3 - 2 to 4 times a month 4 - 2 to 3 times a week 5 - 4 or more times a week |
| freq_alcohol SHOW ONLY IF [alcohol=2 \| alcohol=3 \| alcohol=4 \| alcohol=5] | Average number of ingested alcoholic beverages per drinking day | Factor | 1 - 1 or 2 2 - 3 or 4  3 - 5 or 6 4 - 7 a 9 5 - 10 or more drinks |
| exercise | Participant exercises 30 minutes or more daily and 3 or more time weekly before the RT-PCR COVID-19 test | Factor | 0 - No 1 - Yes |
| **B2. Clinical status before COVID-19** | | | |
| pre_COVID | Previous diagnosis of COVID-19 | Factor | 1 - No 2 - Yes 3 - Unknown |
| prev_COVID_date SHOW ONLY IF [pre_COVID=2] | Diagnosis date of previous COVID-19 infection | Date |  |
| pre_hypertension | Previous diagnosis of hypertension | Factor | 1 - No 2 - Yes 3 - Unknown |
| pre_diabetes | Previous diabetes diagnosis | Factor | 1 - No 2 - Yes 3 - Unknown |
| pre_cholesterol | Previous diagnosis of high cholesterol | Factor | 1 - No 2 - Yes 3 - Unknown |
| pre_asthma | Previous asthma diagnosis | Factor | 1 - No 2 - Yes 3 - Unknown |
| pre_bronchitis | Previous chronic bronchitis or pulmonary emphysema | Factor | 1 - No 2 - Yes 3 - Unknown |
| pre_pulm_fib | Previous pulmonary fibrosis | Factor | 1 - No 2 - Yes 3 - Unknown |
| pre_heart_failure | Previous heart failure | Factor | 1 - No 2 - Yes 3 - Unknown |
| pre_reflux | Previous reflux disease diagnosis | Factor | 1 - No 2 - Yes 3 - Unknown |
| pre_psycho | Previous diagnosis of psychological disorders | Factor | 1 - No 2 - Yes 3 - Unknown |
| pre_myocard_infarc | Previous acute myocardial infarction | Factor | 1 - No 2 - Yes 3 - Unknown |
| pre_stroke | Previous hemorrhagic/ischemic stroke | Factor | 1 - No 2 - Yes 3 - Unknown |
| pre_thrombosis | Previous deep vein thrombosis | Factor | 1 - No 2 - Yes 3 - Unknown |
| pre_pulm_thrombo | Previous pulmonary thromboembolism | Factor | 1 - No 2 - Yes 3 - Unknown |
| height | Participant's height (in meters) | Numeric |  |
| weight_today | Participant's weight (in kgs) at the time of the interview | Numeric |  |
| change_weight | Change in the participant's weight compared with test date | Factor | 1 - No 2 - Yes 3 - Unknown |
| weight_before SHOW ONLY IF [change_weight=2] | Participant's weight (in kgs) at the time of the test | Numeric |  |
| **B3. Symptoms and level of care at the time of the RT-PCR test** | | | |
| pre_cough | Persistent cough or worsening of usual cough at the time of the test | Factor | 1 - No 2 - Yes 3 - Unknown |
| pre_breath | Difficulty breathing at the time of the test | Factor | 1 - No 2 - Yes 3 - Unknown |
| pre_runny_nose | Runny nose at the time of the test | Factor | 1 - No 2 - Yes 3 - Unknown |
| pre_sore_throat | Sore throat at the time of the test | Factor | 1 - No 2 - Yes 3 - Unknown |
| pre_chest | Chest pain at the time of the test | Factor | 1 - No 2 - Yes 3 - Unknown |
| pre_stomach | Stomach/abdominal at the time of the test | Factor | 1 - No 2 - Yes 3 - Unknown |
| pre_vomit | Vomit or nausea at the time of the test | Factor | 1 - No 2 - Yes 3 - Unknown |
| pre_diarrhea | Diarrhea at the time of the test | Factor | 1 - No 2 - Yes 3 - Unknown |
| pre_fever | Fever (≥38º) at the time of the test | Factor | 1 - No 2 - Yes 3 - Unknown |
| pre_chills | Chills at the time of the test | Factor | 1 - No 2 - Yes 3 - Unknown |
| pre_headache | Headache at the time of the test | Factor | 1 - No 2 - Yes 3 - Unknown |
| pre_joint_pain | Joint pain at the time of the test | Factor | 1 - No 2 - Yes 3 - Unknown |
| pre_myalgia | Muscle pain in the entire body at the time of the test | Factor | 1 - No 2 - Yes 3 - Unknown |
| pre_smell | Change in smell at the time of the test | Factor | 1 - No 2 - Yes 3 - Unknown |
| pre_taste | Change in taste at the time of the test | Factor | 1 - No 2 - Yes 3 - Unknown |
| pre_fatigue | Tiredness or lack of strenght at the time of the test | Factor | 1 - No 2 - Yes 3 - Unknown |
| pre_scale_fatigue SHOW ONLY IF [pre_fatigue=2] | Visual analogue scale for the intensity of the tiredness or lack of strenght at the time of the test | Numeric | 0-10 |
| pre_dyspnea | mMRC (Modified Medical Research Council) Dyspnea Scale at the time of the test | Factor | 1 - Dyspnea only with strenuous exercise 2 - Dyspnea when hurrying or walking up a slight hill 3 - Walks slower than people of the same age because of dyspnea or has to stop for breath when walking at own pace 4 - Stops for breath after walking 100 yards (91 m) or after a few minutes 5 - Too dyspneic to leave house or breathless when dressing |
| level_care SHOW ONLY IF [covid_test=1] | Level of care obtained during the disease | Factor | 1 - Did not seek care 2 - Went to the health centre or clinic for an appointment or actively sought an appointment by telephone  3 - Went to the emergency service 4 - Was hospitalised 5 - Was admitted to intensive care 99 - Not applicable |
| qol_mob_pre | Mobility dimension of the EQ-ED-5L scale at the time of the test | Factor | 1 - No problems in walking  2 - Slight problems in walking  3 - Moderate problems in walking  4 - Severe problems in walking  5 - Unable to walk |
| qol_care_pre | Self-care dimension of the EQ-ED-5L scale at the time of the test | Factor | 1 - No problems washing or dressing yourself 2 - Slight problems washing or dressing yourself 3 - Moderate problems washing or dressing yourself 4 - Severe problems washing or dressing yourself 5 - Unable to wash or dress yourself |
| qol_act_pre | Usual activities dimension of the EQ-ED-5L scale at the time of the test | Factor | 1 - No problems doing your usual activities 2 - Slight problems doing your usual activities 3 - Moderate problems doing your usual activities 4 - Severe problems doing your usual activities 5 - Unable to do your usual activities |
| qol_pain_pre | Pain/Discomfort dimension of the EQ-ED-5L scale at the time of the test | Factor | 1 - No pain or discomfort 2 - Slight pain or discomfort 3 - Moderate pain or discomfort 4 - Severe pain or discomfort 5 - Extreme pain or discomfort |
| qol_anx_pre | Anxiety/Depression dimension of the EQ-ED-5L scale at the time of the test | Factor | 1 - Not anxious or depressed 2 - Slightly anxious or depressed 3 - Moderately anxious or depressed 4 - Severely anxious or depressed 5 - Extremely anxious or depressed |
| eq_vas_pre | EQ-VAS at the time of the test | Numeric | 0-100 |
| **B4. Symptoms, health status and vaccination at the moment of the interview** | | | |
| recovered_COVID SHOW ONLY IF [covid_test=1] | Full recovery from the COVID-19 disease | Factor | 1 - Totally disagree 2 - Disagree 3 - Neither agree nor disagree 4 - Agree 5 - Totally agree 99 - Not applicable |
| now_cough | Persistent cough or worsening of usual cough in the preceding 7 days of the interview | Factor | 1 - No 2 - Yes 3 - Unknown |
| now_breath | Shortness of breath/breathlessness in the preceding 7 days of the interview | Factor | 1 - No 2 - Yes 3 - Unknown |
| now_runny_nose | Runny nose in the preceding 7 days of the interview | Factor | 1 - No 2 - Yes 3 - Unknown |
| now_sore_throat | Sore throat in the preceding 7 days of the interview | Factor | 1 - No 2 - Yes 3 - Unknown |
| now_chest | Chest pain in the preceding 7 days of the interview | Factor | 1 - No 2 - Yes 3 - Unknown |
| now_stomach | Stomach/abdominal pain in the preceding 7 days of the interview | Factor | 1 - No 2 - Yes 3 - Unknown |
| now_vomit | Vomit or nausea in the preceding 7 days of the interview | Factor | 1 - No 2 - Yes 3 - Unknown |
| now_diarrhea | Diarrhea in the preceding 7 days of the interview | Factor | 1 - No 2 - Yes 3 - Unknown |
| now_fever | Fever (≥38º) in the preceding 7 days of the interview | Factor | 1 - No 2 - Yes 3 - Unknown |
| now_chills | Chills in the preceding 7 days of the interview | Factor | 1 - No 2 - Yes 3 - Unknown |
| now_headache | Headache in the preceding 7 days of the interview | Factor | 1 - No 2 - Yes 3 - Unknown |
| now_joint_pain | Joint pain in the preceding 7 days of the interview | Factor | 1 - No 2 - Yes 3 - Unknown |
| now_myalgia | Muscle pain in the entire body in the preceding 7 days of the interview | Factor | 1 - No 2 - Yes 3 - Unknown |
| now_smell | Change in smell in the preceding 7 days of the interview | Factor | 1 - No 2 - Yes 3 - Unknown |
| now_taste | Change in taste in the preceding 7 days of the interview | Factor | 1 - No 2 - Yes 3 - Unknown |
| now_fatigue | Tiredness or lack of strenght in the preceding 7 days of the interview | Factor | 1 - No 2 - Yes 3 - Unknown |
| now_pain_breath | Pain breathing in the preceding 7 days of the interview | Factor | 1 - No 2 - Yes 3 - Unknown |
| now_palpitations | Palpitations in the preceding 7 days of the interview | Factor | 1 - No 2 - Yes 3 - Unknown |
| now_loss_appetite | Loss of apetite in the preceding 7 days of the interview | Factor | 1 - No 2 - Yes 3 - Unknown |
| now_constipation | Constipation in the preceding 7 days of the interview | Factor | 1 - No 2 - Yes 3 - Unknown |
| now_pass_urine | Problems passing urine in the preceding 7 days of the interview | Factor | 1 - No 2 - Yes 3 - Unknown |
| now_erectile SHOW ONLY IF [sex=1] | Erectile dysfunction or unusual difficulties in achieving or maintaining an erection in the preceding 7 days of the interview | Factor | 1 - No 2 - Yes 3 - Unknown 99 - Not applicable |
| now_menstruation SHOW ONLY IF [sex=2 & age<55] | Changes in the menstrual cycle in the preceding 7 days of the interview | Factor | 1 - No 2 - Yes 3 - Unknown 99 - Not applicable |
| now_swollen_ankle | Swollen ankle/s in the preceding 7 days of the interview | Factor | 1 - No 2 - Yes 3 - Unknown |
| now_balance | Problems with balance in the preceding 7 days of the interview | Factor | 1 - No 2 - Yes 3 - Unknown |
| now_feeling | Cannot feel one side of the body or face in the preceding 7 days of the interview | Factor | 1 - No 2 - Yes 3 - Unknown |
| now_tingling | Tingling feeling/“pins and needles“ in the preceding 7 days of the interview | Factor | 1 - No 2 - Yes 3 - Unknown |
| now_faint | Fainting in the preceding 7 days of the interview | Factor | 1 - No 2 - Yes 3 - Unknown |
| now_seizure | Seizures in the preceding 7 days of the interview | Factor | 1 - No 2 - Yes 3 - Unknown |
| now_tremors | Tremor/shakiness in the preceding 7 days of the interview | Factor | 1 - No 2 - Yes 3 - Unknown |
| now_diff_swallow | Problems swallowing in the preceding 7 days of the interview | Factor | 1 - No 2 - Yes 3 - Unknown |
| now_diff_chew | Problems chewing in the preceding 7 days of the interview | Factor | 1 - No 2 - Yes 3 - Unknown |
| now_buzz | Ringing in ear in the preceding 7 days of the interview | Factor | 1 - No 2 - Yes 3 - Unknown |
| now_insomnia | Insomnia in the preceding 7 days of the interview | Factor | 1 - No 2 - Yes 3 - Unknown |
| now_rash | Skin rash in the preceding 7 days of the interview | Factor | 1 - No 2 - Yes 3 - Unknown |
| now_confusion | Confusion/lack of concentration in the preceding 7 days of the interview | Factor | 1 - No 2 - Yes 3 - Unknown |
| now_diff_memory | Difficulty remembering in the preceding 7 days of the interview | Factor | 1 - No 2 - Yes 3 - Unknown |
| now_scale_fatigue SHOW ONLY IF [now_fatigue=2] | Visual analogue scale for the intensity of the tiredness or lack of strenght at the time of the test | Numeric | 0-10 |
| seek_care SHOW ONLY IF [now_cough=2 \| now_breath=2 \| now_runny_nose=2 \| now_sore_throat=2 \| now_chest=2 \| now_stomach=2 \| now_vomit=2 \| now_diarrhea=2 \| now_fever=2 \| now_chills=2 \| now_headache=2 \| now_joint_pain=2 \| now_myalgia=2 \| now_smell=2 \| now_taste=2 \| now_fatigue=2 \| now_pain_breath=2 \| now_palpitations=2 \| now_loss_appetite=2 \| now_constipation=2 \| now_pass_urine=2 \| now_erectile=2 \| now_menstruation \| now_swollen_ankle=2 \| now_balance=2 \| now_feeling=2 \| now_tingling=2 \| now_faint=2 \| now_seizure=2 \| now_tremors=2 \| now_diff_swallow=2 \| now_diff_chew=2 \| now_buzz=2 \| now_insomnia=2 \| now_rash=2 \| now_confusion=2 \| now_diff_memory=2] | Participant's sought care for any of the symptoms felt | Factor | 0 - No 1 - Yes |
| PCC_diag SHOW ONLY IF [seek_care=1] | Participant received a diagnosis of post-COVID-19 condition | Factor | 0 - No 1 - Yes |
| work_impact SHOW ONLY IF [(now_cough=2 \| now_breath=2 \| now_runny_nose=2 \| now_sore_throat=2 \| now_chest=2 \| now_stomach=2 \| now_vomit=2 \| now_diarrhea=2 \| now_fever=2 \| now_chills=2 \| now_headache=2 \| now_joint_pain=2 \| now_myalgia=2 \| now_smell=2 \| now_taste=2 \| now_fatigue=2 \| now_pain_breath=2 \| now_palpitations=2 \| now_loss_appetite=2 \| now_constipation=2 \| now_pass_urine=2 \| now_erectile=2 \| now_menstruation \| now_swollen_ankle=2 \| now_balance=2 \| now_feeling=2 \| now_tingling=2 \| now_faint=2 \| now_seizure=2 \| now_tremors=2 \| now_diff_swallow=2 \| now_diff_chew=2 \| now_buzz=2 \| now_insomnia=2 \| now_rash=2 \| now_confusion=2 \| now_diff_memory=2) & (current_job=1 \| current_job=4)] | Extent symptoms felt have affected work productivity in the preceding 7 days | Numeric | 0-10 |
| act_impact SHOW ONLY IF [(now_cough=2 \| now_breath=2 \| now_runny_nose=2 \| now_sore_throat=2 \| now_chest=2 \| now_stomach=2 \| now_vomit=2 \| now_diarrhea=2 \| now_fever=2 \| now_chills=2 \| now_headache=2 \| now_joint_pain=2 \| now_myalgia=2 \| now_smell=2 \| now_taste=2 \| now_fatigue=2 \| now_pain_breath=2 \| now_palpitations=2 \| now_loss_appetite=2 \| now_constipation=2 \| now_pass_urine=2 \| now_erectile=2 \| now_menstruation \| now_swollen_ankle=2 \| now_balance=2 \| now_feeling=2 \| now_tingling=2 \| now_faint=2 \| now_seizure=2 \| now_tremors=2 \| now_diff_swallow=2 \| now_diff_chew=2 \| now_buzz=2 \| now_insomnia=2 \| now_rash=2 \| now_confusion=2 \| now_diff_memory=2) & (current_job=1 \| current_job=4)] | Extent symptoms felt have affected usual daily non-work activities in the preceding 7 days | Numeric | 0-10 |
| now_dyspnea | mMRC (Modified Medical Research Council) Dyspnea Scale at the time of the interview | Factor | 1 - Dyspnea only with strenuous exercise 2 - Dyspnea when hurrying or walking up a slight hill 3 - Walks slower than people of the same age because of dyspnea or has to stop for breath when walking at own pace 4 - Stops for breath after walking 100 yards (91 m) or after a few minutes 5 - Too dyspneic to leave house or breathless when dressing |
| qol_mob_now | Mobility dimension of the EQ-ED-5L scale at the time of the interview | Factor | 1 - No problems in walking  2 - Slight problems in walking  3 - Moderate problems in walking  4 - Severe problems in walking  5 - Unable to walk |
| qol_care_now | Self-care dimension of the EQ-ED-5L scale at the time of the interview | Factor | 1 - No problems washing or dressing yourself 2 - Slight problems washing or dressing yourself 3 - Moderate problems washing or dressing yourself 4 - Severe problems washing or dressing yourself 5 - Unable to wash or dress yourself |
| qol_act_now | Usual activities dimension of the EQ-ED-5L scale at the time of the interview | Factor | 1 - No problems doing your usual activities 2 - Slight problems doing your usual activities 3 - Moderate problems doing your usual activities 4 - Severe problems doing your usual activities 5 - Unable to do your usual activities |
| qol_pain_now | Pain/Discomfort dimension of the EQ-ED-5L scale at the time of the interview | Factor | 1 - No pain or discomfort 2 - Slight pain or discomfort 3 - Moderate pain or discomfort 4 - Severe pain or discomfort 5 - Extreme pain or discomfort |
| qol_anx_now | Anxiety/Depression dimension of the EQ-ED-5L scale at the time of the interview | Factor | 1 - Not anxious or depressed 2 - Slightly anxious or depressed 3 - Moderately anxious or depressed 4 - Severely anxious or depressed 5 - Extremely anxious or depressed |
| eq_vas_now | EQ-VAS at the time of the interview | Numeric | 0-100 |
| now_COVID | Diagnosis of COVID-19 since PCR COVID-19 test | Factor | 1 - No 2 - Yes 3 - Unknown |
| now_COVID_date SHOW ONLY IF [now_COVID=2] | Diagnosis date of COVID-19 infection since PCR COVID-19 test | Date |  |
| now_hypertension SHOW ONLY IF [pre_hypertension=1] | Hypertension diagnosis since PCR COVID-19 test | Factor | 1 - No 2 - Yes 3 - Unknown |
| now_diabetes SHOW ONLY IF [pre_diabetes=1] | Diabetes diagnosis since PCR COVID-19 test | Factor | 1 - No 2 - Yes 3 - Unknown |
| now_cholesterol SHOW ONLY IF [pre_cholesterol=1] | High cholesterol diagnosis since PCR COVID-19 test | Factor | 1 - No 2 - Yes 3 - Unknown |
| now_asthma SHOW ONLY IF [pre_asthma=1] | Asthma diagnosis since PCR COVID-19 test | Factor | 1 - No 2 - Yes 3 - Unknown |
| now_bronchitis SHOW ONLY IF [pre_bronchitis=1] | Chronic bronchitis or pulmonary emphysema diagnosis since PCR COVID-19 test | Factor | 1 - No 2 - Yes 3 - Unknown |
| now_myocard_infarc SHOW ONLY IF [pre_myocard_infarc=1] | Acute myocardial infarction since PCR COVID-19 test | Factor | 1 - No 2 - Yes 3 - Unknown |
| now_stroke SHOW ONLY IF [pre_stroke=1] | Hemorrhagic/ischemic stroke since PCR COVID-19 test | Factor | 1 - No 2 - Yes 3 - Unknown |
| now_thrombosis SHOW ONLY IF [pre_thrombosis=1] | Deep vein thrombosis since PCR COVID-19 test | Factor | 1 - No 2 - Yes 3 - Unknown |
| now_pulm_thrombo SHOW ONLY IF [pre_pulm_thrombo=1] | Pulmonary thromboembolism since PCR COVID-19 test | Factor | 1 - No 2 - Yes 3 - Unknown |
| other_diag | Received any other diagnosis since PCR COVID-19 test | Factor | 0 - No 1 - Yes |
| other_diag_text SHOW ONLY IF [other_diag=1] | Diagnosis received since PCR COVID-19 test | Text |  |
| vac_covid | Participant received a vaccine against COVID-19 | Factor | 0 - No 1 - Yes |
| doses_covid SHOW ONLY IF [vac_covid=1] | Number of doses the participant received | Numeric | 1-3 |
| first_date_vac SHOW ONLY IF [doses_covid=1] | Date of first vaccine dose against COVID-19 | Date |  |
| first_brand_vac SHOW ONLY IF [doses_covid=1] | Brand of the first vaccine against COVID-19 received | Factor | 1 - Pfizer/Comirnaty 2 - Moderna/Spikevax 3 - Astrazeneca 4 - Janssen 5 - Other |
| second_date_vac SHOW ONLY IF [doses_covid≥2] | Date of the second vaccine dose against COVID-19 | Date |  |
| second_brand_vac SHOW ONLY IF [doses_covid≥2] | Brand of the second vaccine against COVID-19 received | Factor | 1 - Pfizer/Comirnaty 2 - Moderna/Spikevax 3 - Astrazeneca 4 - Other |
| third_date_vac SHOW ONLY IF [doses_covid=3] | Date of the third vaccine dose against COVID-19 | Date |  |
| third_brand_vac SHOW ONLY IF [doses_covid=3] | Brand of the third vaccine against COVID-19 received | Factor | 1 - Pfizer/Comirnaty 2 - Moderna/Spikevax 3 - Other |
| flu_vac | Participant received the flu vaccine in the past year | Factor | 0 - No 1 - Yes |
| antipneumoc | Received the antipneumococcal vaccine in the last 5 years | Factor | 0 - No 1 - Yes |
| **B5. Mental health scales** | | | |
| phq9_1 | How often has the participant had little interest or pleasure in doing things in the past two weeks | Factor | 1 - Never 2 - Several days  3 - More than half the days 4 - Nearly every day |
| phq9_2 | How often has the participant been feeling down, depressed, or hopeless in the past two weeks | Factor | 1 - Never 2 - Several days  3 - More than half the days 4 - Nearly every day |
| screen_phq2 | Categorization of the sum of the phq9_1 and phq9_2 | Factor | 1 - <3 2 - ≥3 |
| phq9_3 SHOW ONLY IF [screen_phq2=2] | How often has the participant had trouble falling or staying asleep, or sleeping too much in the past two weeks | Factor | 1 - Never 2 - Several days  3 - More than half the days 4 - Nearly every day 99 - Not applicable |
| phq9_4 SHOW ONLY IF [screen_phq2=2] | How often has the participant been feeling tired or having little energy in the past two weeks | Factor | 1 - Never 2 - Several days  3 - More than half the days 4 - Nearly every day 99 - Not applicable |
| phq9_5 SHOW ONLY IF [screen_phq2=2] | How often has the participant had poor appetite or overeating in the past two weeks | Factor | 1 - Never 2 - Several days  3 - More than half the days 4 - Nearly every day 99 - Not applicable |
| phq9_6 SHOW ONLY IF [screen_phq2=2] | How often has the participant have been feeling bad about themselves — or that they were a failure or have let themselves or their family down in the past two weeks | Factor | 1 - Never 2 - Several days  3 - More than half the days 4 - Nearly every day 99 - Not applicable |
| phq9_7 SHOW ONLY IF [screen_phq2=2] | How often has the participant had trouble concentrating on things, such as reading the newspaper or watching television in the past two weeks | Factor | 1 - Never 2 - Several days  3 - More than half the days 4 - Nearly every day 99 - Not applicable |
| phq9_8 SHOW ONLY IF [screen_phq2=2] | How often has the participant have been moving or speaking so slowly that other people could have noticed, or was so fidgety or restless that they have been moving a lot more than usual in the past two weeks | Factor | 1 - Never 2 - Several days  3 - More than half the days 4 - Nearly every day 99 - Not applicable |
| phq9_9 SHOW ONLY IF [screen_phq2=2] | How often has the participant had thoughts that they were be better off dead, or thoughts of hurting themselves in some way in the past two weeks | Factor | 1 - Never 2 - Several days  3 - More than half the days 4 - Nearly every day 99 - Not applicable |
| phq9_score | Categorization of the PHQ9 scale | Factor | 1 - None (0 – 4) 2 - Mild (5 – 9) 3 - Moderate (10 – 14) 4 - Moderately severe (15 – 19) 5 - Severe (20 – 27) |
| gad7_1 | How often has the participant have been feeling nervous, anxious, or on edge in the past two weeks | Factor | 1 - Never 2 - Several days  3 - More than half the days 4 - Nearly every day |
| gad7_2 | How often has the participant not been able to stop or control worrying in the past two weeks | Factor | 1 - Never 2 - Several days  3 - More than half the days 4 - Nearly every day |
| screen_gad2 | Categorization of the sum of the gad7_1 and gad7_2 | Factor | 1 - <3 2 - ≥3 |
| gad7_3 SHOW ONLY IF  [screen_gad2=2] | How often has the participant been worrying too much about different things in the past two weeks | Factor | 1 - Never 2 - Several days  3 - More than half the days 4 - Nearly every day 99 - Not applicable |
| gad7_4 SHOW ONLY IF  [screen_gad2=2] | How often has the participant had trouble relaxing in the past two weeks | Factor | 1 - Never 2 - Several days  3 - More than half the days 4 - Nearly every day 99 - Not applicable |
| gad7_5 SHOW ONLY IF  [screen_gad2=2] | How often has the participant being so restless that it's hard to sit still in the past two weeks | Factor | 1 - Never 2 - Several days  3 - More than half the days 4 - Nearly every day 99 - Not applicable |
| gad7_6 SHOW ONLY IF  [screen_gad2=2] | How often has the participant became easily annoyed or irritable in the past two weeks | Factor | 1 - Never 2 - Several days  3 - More than half the days 4 - Nearly every day 99 - Not applicable |
| gad7_7 SHOW ONLY IF  [screen_gad2=2] | How often has the participant feeling afraid as if something awful might happen in the past two weeks | Factor | 1 - Never 2 - Several days  3 - More than half the days 4 - Nearly every day 99 - Not applicable |
| gad7_score | Categorization of the GAD7 scale | Factor | 1 - Minimal (0 – 4) 2 - Mild (5 – 9) 3 - Moderate (10 – 14) 4 - Severe (≥15) |
| pandemic_trauma | Participant considered the pandemic a traumatic or frightening event | Factor | 0 - No 1 - Yes |
| ptsd_nightmare SHOW ONLY IF [pandemic_trauma=1] | Has the participant had nightmares about the pandemic or thought about the pandemic even when they did not want in the previous month | Factor | 0 - No 1 - Yes 99 - Not applicable |
| ptsd_think SHOW ONLY IF [pandemic_trauma=1] | Has the participant tried hard not to think about the pandemic or went out of their way to avoid situations that reminded them of the pandemic in the previous month | Factor | 0 - No 1 - Yes 99 - Not applicable |
| ptsd_startled SHOW ONLY IF [pandemic_trauma=1] | Has the participant been constantly on guard, watchful, or easily startled in the previous month | Factor | 0 - No 1 - Yes 99 - Not applicable |
| ptsd_numb SHOW ONLY IF [pandemic_trauma=1] | Has the participant felt numb or detached from people, activities, or surroundings in the previous month | Factor | 0 - No 1 - Yes 99 - Not applicable |
| ptsd_guilt SHOW ONLY IF [pandemic_trauma=1] | Has the participant felt guilty or unable to stop blaming themselves or others for the pandemic or any problems the pandemic may have caused in the previous month | Factor | 0 - No 1 - Yes 99 - Not applicable |
