# Supplementary material for: LOCUS (LOng Covid–Understanding Symptoms, events and use of services in Portugal): A three-component study protocol
Source: PLoS One. 2023 Apr 26;18(4):e0285051. doi: 10.1371/journal.pone.0285051 (PMC10132590; doi:10.1371/journal.pone.0285051)
Supplement: S2 Table — (DOCX) [file pone.0285051.s002.docx]

**Supporting information – S2 Table**

**S2 Table.** Ethics approval of the three components of the study**.**

| **Institution** | **Component(s) evaluated** | **Registry number** | **Date** |
| --- | --- | --- | --- |
| Comissão de Ética para a Saúde da ARSLVT (*Ethics Committee for Health of the Regional Health Administration of Lisbon and Tagus Valley*) | “Physical and mental symptoms following COVID-19” and "Treating and living with Post COVID-19 Condition" | 2151/CES/2022 | March, 11^th^ 2022 |
| Comissão de Ética do Hospital de Cascais (*Ethics Committee of the Cascais Hospital*) | “Cardiovascular and respiratory events following COVID-19” | 10/CE | May, 16^th^ 2022 |
| Comissão de Ética para a Saúde do Centro Hospitalar e Universitário de Coimbra (*Ethics Committee for Health of the Coimbra Hospital and University Centre*) | “Cardiovascular and respiratory events following COVID-19” | 240/CES | June, 2^nd^ 2022 |
| Comissão de Ética para a Saúde do Serviço de Saúde da Região Autónoma da Madeira (*Ethics Committee for Health of the Madeira Autonomous Region Health Service)* | “Cardiovascular and respiratory events following COVID-19” | S.22001906 | April, 29^th^ 2022 |
| Comissão de Ética para a Saúde do Centro Hospitalar da Póvoa de Varzim e Vila do Conde (*Ethics Committee for Health of the Póvoa de Varzim and Vila do Conde Hospital Hospital Centre*) | “Cardiovascular and respiratory events following COVID-19” | NA | May, 4^th^ 2022 |
| Comissão de Ética para a Saúde do Centro Hospitalar Tondela - Viseu (*Ethics Committee for Health of the Tondela - Viseu Hospital Centre*) | “Cardiovascular and respiratory events following COVID-19” | 04/22/04/2022 | April, 22^nd^ 2022 |
